# Editorial: Biological Control Systems and Disease Modeling

**DOI:** 10.3389/fbioe.2021.677976

**Published:** 2021-04-09

**Authors:** Babatunde Ogunnaike, Julio R. Banga, David Bogle, Robert Parker

**Affiliations:** ^1^Department of Chemical & Bimolecular Engineering, University of Delaware, Newark, NJ, United States; ^2^Consejo Superior de Investigaciones Científicas (CSIC), Madrid, Spain; ^3^Department of Chemical Engineering, University College London, London, United Kingdom; ^4^Department of Chemical and Petroleum Engineering, University of Pittsburgh, Pittsburgh, PA, United States

**Keywords:** systems biology, mathematical modeling, biological control systems, precision medicine, disease modeling

## Introduction

The mammalian organism maintains stable, efficient, and “near-optimal” performance and *homeostasis* in the face of external and internal perturbations via distinct biological systems ranging from the large-scale *physiological* (nervous, endocrine, immune, circulatory, respiratory, etc.), to the *cellular* (growth and proliferation regulation, DNA damage repair, etc.), and the *sub-cellular* (gene expression, protein synthesis, metabolite regulation, etc). “Biological Control Systems,” the application of control theory and practice to biological systems, arises from a control engineering perspective of the function, organization, and coordination of these multi-scale biological systems and the control mechanisms that enable them to carry out their functions effectively. A direct consequence of this engineering perspective is that many diseases are seen as arising when one (or more) of these biological control systems malfunctions or fails completely. For example, hypertension results from a malfunctioning blood pressure control system, hypocalcemia from a malfunctioning calcium homeostasis system, and Type 1 diabetes from a failure of the blood glucose control system. A natural corollary therefore is that appropriate treatment regimens consist of ways of restoring (for example via pharmaceutical drugs) the functions lost by the malfunctioning components, or, where full functional restoration is not possible, the introduction of external means of supplementing or replacing the malfunctioning biological component (for example, the “artificial pancreas” for treating Type-I diabetes). Such a perspective places emphasis on a rigorous *quantitative* approach to three tasks: (i) the analysis of biological systems for insight; (ii) the identification of the root cause of pathologies and potential treatment targets; and (iii) the rational design (and implementation) of effective interventions.

## Overview

This collection of twenty papers seeks to provide—under a single cover—a broad spectrum of research results showcasing this quantitative perspective of the physiological processes that subtend life, the diseases that occur when these systems malfunction, and the design of effective treatments for these diseases, specifically using mathematical modeling and principles of systems engineering. For obvious reasons of space limitation, the collection is naturally representative rather than exhaustive.

Nevertheless, the papers address a wide variety of diseases including non-alcoholic fatty liver disease; hepatic steatosis; cancers of various types (liver, blood, breast, leukemia, papillary renal cell carcinoma); immune system dysregulation; neurodegeneration diseases; and such “classics” as diabetes and HIV. Some of the papers focus on entire physiological processes, such as the cardiovascular system, or the specialized process of parturition—how babies are delivered vaginally following pregnancy; others focus on cellular and signaling sub-processes that subtend the physiological manifestation of diseases. Viewed from another vantage point, some papers are concerned with how the physiological control systems function endogenously while others focus on how to design effective external interventions, such as designing optimal personalized treatments for blood cancer patients, or the use of the engineering technique of model predictive control (MPC) to control macrophage polarization. Also, the approaches represented in this collection cover the entire length scale from the genetic and cellular to the tissue and physiological. Also, while some of the results are strictly theoretical, a significant number of these results have been validated experimentally, while some are entirely experimental in nature. On many levels, therefore, the papers are sufficiently complementary to provide a good representation of the broad and expanding landscape of quantitative biomedicine viewed through the lens of biological control systems.

## Unifying Theme

While the diseases and physiological processes encountered here are richly diverse, the common unifying thread holding the 20 papers together is the use of a mathematical model—of one type or another—to quantify the phenomenon in question and employing such a quantitative description to answer a wide variety of questions for many different applications. For example, in this collection: mathematical models are used for disease diagnosis; for rational identification of treatment targets; for design, analysis, and implementation of optimal treatment regimens; for prognosis; and even as a surrogate for clinical trials. In addition, the papers collectively illustrate the diversity of mathematical models *themselves* and the versatility with which they can be developed, validated, deployed, and utilized, depending on the actual application in question. Consequently, in addition to the standard ordinary differential equation (ODE) models with which most readers will likely be familiar, one will find in this collection, such exemplars of mechanistic models as partial differential equation models used to capture spatial variations when these are important, or steady state metabolic flux models. At the other end of the spectrum, one will also find models based entirely on data, employing techniques such as support vector machines, data mining, and machine learning to develop the appropriate data-based model best suited to the problem at hand. Hybrid models, which occupy the vast domain in between these two ends of the modeling spectrum, are also represented here. This class of models arise by combining mechanistic principles appropriately with empirical data in proportions typically dictated by which is more readily available in the desired amount—first principles knowledge, or data. There are also in this collection a handful of applications of other techniques with which the average reader may not be familiar, such as fractals, or agent-based models, presented here as systematic frameworks best suited to studying complex and heterogenous systems (e.g., tumor microenvironments and microbiomes) that would otherwise be virtually impossible to study systematically. The variety of mathematical model forms represented in this collection, when overlaid onto the myriad applications and diseases to which they have been applied, underscores the depth of penetration of mathematical modeling into modern physiology and medicine, and the effectiveness of quantitative techniques in providing heretofore unimaginable solutions.

## Key Implications of Results

With the exception of the lone review/perspective paper (on image-based computational modeling for non-invasive acquisition of the crucial measurements required for personalized cardiovascular medicine), every paper in this collection *by itself* contributes important results that add to the growing consensus of the role of quantitative analysis in the understanding of biological systems at all length scales, the rational deduction of the sources and emergence of diseases, and the determination of *optimal* treatment regimens—how best to intervene to treat these diseases while minimizing side effects. Taken together as an ensemble, here are some of the most significant implications of the results in this collection:

- *Growing indispensability of mathematical modeling in biomedicine*: The importance and scope of mathematical modeling in understanding diseases as complex systems, and in designing rational treatment regimens, continues to grow, underscored and exemplified by the novel implementation of a virtual clinical trial in HER2-Negative Breast Cancer, with major implications for personalized medicine at reasonable cost.
- *Novel extraction and utilization of germane actionable information from data*: Beyond the traditional role as the basis for data-based model development, appropriately acquired data sets and the judicious extraction of the information contained therein have the potential to enable modern biomedicine in unprecedented ways, ranging from early—and reliable—diagnosis of liver cancer; more accurate prognosis of papillary renal cell carcinoma; and the identification of effective therapeutic targets based on gene networks deduced from gene expression data.
- *Effective disease treatment as a control problem*: The implementation of appropriate treatments for some diseases can be formulated as an engineering control problem, which then allows one to invoke principles and established results from that engineering field and modify and/or extend them appropriately for use in designing optimal treatment regimens for diseases. The implementation of such an approach is exemplified here with specific applications ranging from theoretical analysis of drug resistance in cancer chemotherapy, leading to à*-priori* design of patient specific optimal therapies; controlling macrophage polarization using model predictive control (MPC); or personalized optimization of blood cancer treatment.

In conclusion, we believe that the message in this collection of papers is both relevant and timely. With attention currently focused on precision (more appropriately *personalized*) medicine, the approaches discussed and illustrated by these papers should contribute significantly to how the grand vision of personalizing disease diagnosis and treatment will be realized in the future. Consequently, we are pleased to present this collection to the community with the belief that it will be useful to the beginner (to obtain a broad overview of the evolving landscape) as well as to the expert who might find therein a useful stepping stone to the next contribution that will further extend the frontier of knowledge in the subject of biological control systems.

## Author Contributions

All authors listed have made a substantial, direct and intellectual contribution to the work, and approved it for publication.

## Conflict of Interest

The authors declare that the research was conducted in the absence of any commercial or financial relationships that could be construed as a potential conflict of interest.

